# 6-Nitro-2-(3-hydroxypropyl)-1H-benz[de]isoquinoline-1,3-dione, a potent antitumor agent, induces cell cycle arrest and apoptosis

**DOI:** 10.1186/1756-9966-29-175

**Published:** 2010-12-31

**Authors:** Asama Mukherjee, Sushanta Dutta, Muthiah Shanmugavel, Dilip M Mondhe, Parduman R Sharma, Shashank K Singh, Ajit K Saxena, Utpal Sanyal

**Affiliations:** 1 Department of Anticancer Drug Development, Chittaranjan National Cancer Institute, Kolkata 700026, India; 2 Pharmacology Division, Indian Institute of Integrative Medicine, Canal Road, Jammu-Tawi 180001, India

## Abstract

**Background:**

Anticancer activities of several substituted naphthalimides (1H-benz[de]isoquinoline-1,3-diones) are well documented. Some of them have undergone Phase I-II clinical trials. Presently a series of ten N-(hydroxyalkyl) naphthalimides (compounds **1a-j) **were evaluated as antitumor agents.

**Methods:**

Compounds **1a-j **were initially screened in MOLT-4, HL-60 and U-937 human tumor cell lines and results were compared with established clinical drugs. Cytotoxicities of compounds **1d **and **1i **were further evaluated in a battery of human tumor cell lines and in normal human peripheral blood mononuclear cells. Cell cycle analysis of compound **1i **treated MOLT-4 cells was studied by flow cytometry. Its apoptosis inducing effect was carried out in MOLT-4 and HL-60 cells by flow cytometry using annexin V-FITC/PI double staining method. The activities of caspase-3 and caspase-6 in MOLT-4 cells following incubation with compound **1i **were measured at different time intervals. Morphology of the MOLT-4 cells after treatment with **1i **was examined under light microscope and transmission electron microscope. ^3^H-Thymidine and ^3^H-uridine incorporation in S-180 cells in vitro following treatment with 8 μM concentration of compounds **1d **and **1i **were studied.

**Results:**

6-Nitro-2-(3-hydroxypropyl)-1H-benz[de]isoquinoline-1,3-dione (compound **1i**), has exhibited maximum activity as it induced significant cytotoxicity in 8 out of 13 cell lines employed. Interestingly it did not show any cytotoxicity against human PBMC (IC_50 _value 273 μM). Cell cycle analysis of compound **1i **treated MOLT-4 cells demonstrated rise in sub-G_1 _fraction and concomitant accumulation of cells in S and G_2_/M phases, indicating up-regulation of apoptosis along with mitotic arrest and/or delay in exit of daughter cells from mitotic cycle respectively. Its apoptosis inducing effect was confirmed in flow cytometric study in MOLT-4 and the action was mediated by activation of both caspase 3 and 6. Light and transmission electron microscopic studies corroborated its apoptosis inducing efficacy at a concentration of 10 μM in MOLT-4 cells. Its apoptosis induction was also observed in HL-60 cells to an extent much greater than well known apoptosis inducing agents as camptothecin and cis-platin at 10 μM concentration each. It significantly inhibited DNA and RNA synthesis in S-180.

**Conclusions:**

In essence, compound **1i **showed potential as an antitumor agent.

## Background

Development of an anticancer compound is always a fascinating challenge in the field of cancer chemotherapy. Research is ongoing globally to identify new leads. The anticancer activities of several substituted naphthalimides (1H-benz[de]isoquinoline-1,3-diones) are well documented [1,2]. For example, substituted naphthalimides containing N-(2,2-dimethylaminoethyl) chain best represented by Mitonafide (5-nitro group in the aromatic ring) and Amonafide (5-amino group in the aromatic ring) have been shown to possess significant anticancer activities. Both Mitonafide [3,4] and Amonafide [5,6] have undergone Phase I-II clinical trials with limited success. We have recently reported appreciable antitumor activity of some new compounds belonging to N-(2-chloroethyl)- and N-(3-chloropropyl) naphthalimides [7]. From the literature search, it was found that there was no report, to our knowledge, that describes the anticancer potential of known N-(2-hydroxyethyl) and N-(3-hydroxypropyl) naphthalimides (compounds **1a-j)**. Hence we have undertaken the present study of evaluating their potency. In this report we have documented the findings that shows that 6-nitro-2-(3-hydroxypropyl)-1H-benz[de]isoquinoline-1,3-dione (compound **1i**) is the most active member in the series.

## Materials and methods

### Chemicals and drugs

**Figure 1:**
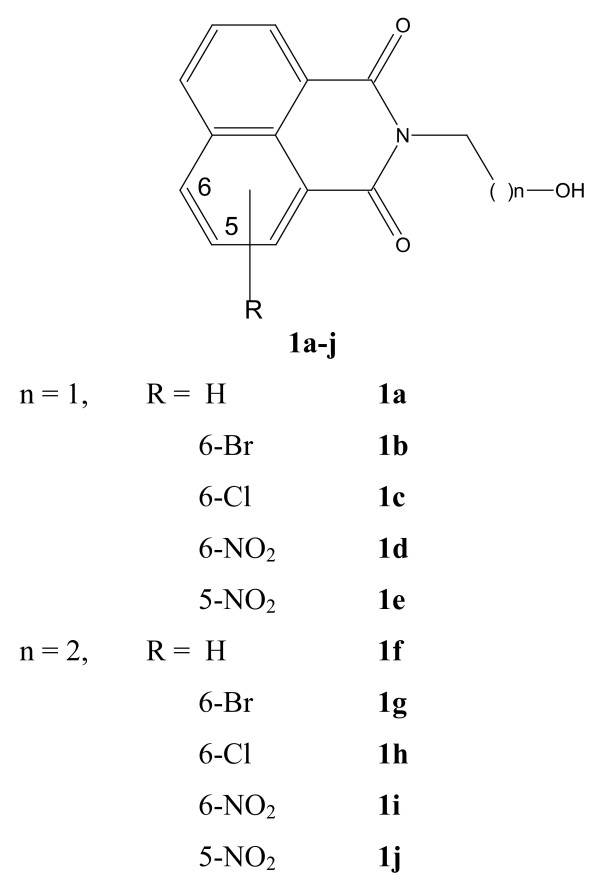
**Chemical structures of compounds 1a-j**.

A total number of ten substituted 2-(2-hydroxyethyl)- and 2-(3-hydroxypropyl)-1H-benz[de]isoquinoline-1,3-diones (compounds **1a-j) **(Figure 1) were prepared following established procedure. Out of these ten compounds, test compound **1i **[8] was most extensively investigated. Mitonafide was received earlier as a gift from Prof. M.F. Brana, University of San Pablo-CEU, Madrid, Spain. Anticancer drugs, propidium iodide and annexin V-FITC detection kit (A2214) were procured from Sigma-Aldrich Corporation, St. Louis, MO, USA.

### Culture of human tumor cell lines

The following human tumor cell lines namely Leukemia: acute lymphoblastic MOLT-4, promyelocytic HL-60; Lymphoma: histiocytic U-937; Breast: MCF-7; Neuroblastoma: IMR-32, SK-N-SH; Colon: 502713, COLO-205, HCT-15, SW-620; Liver: Hep-2; Prostate: DU-145, PC-3 and Lung: A549 obtained either from National Centre of Cell Science (NCCS), Pune, India or National Cancer Institute, Fredrick, MD, USA were used. Cell lines were grown in tissue culture flasks in RPMI-1640 medium with 2 mM glutamine (Invitrogen Corporation, USA) containing 1% antibiotics (100 units penicillin/ml and 100 μg streptomycin/ml, Cambrex Bioscience Inc., USA), pH 7.4, sterilized by filtration and supplemented with 10% heat-inactivated fetal bovine serum (FBS, Invitrogen Corporation, USA) at 37°C in an atmosphere of 5% CO_2_/95% relative humidity in a CO_2 _incubator and routinely sub-cultured. Trypsin (0.02%) was used for dislodging adherent type cells.

### In vitro screening in human tumor cell lines

All the test compounds **1a-j **were initially screened against U-937 and HL-60 cell lines by MTT assay as per standard procedure [9]. Compounds **1d **and **1i **were also screened in MOLT-4 (Table 1). Drug stock solutions (20 mg/ml) were prepared in cell culture DMSO. These were serially diluted with complete growth medium stated above to obtain different drug concentrations [final DMSO concentration was 0.5% highest to 0.001% lowest]. Cells were seeded at 1 × 10^4 ^(U-937), 2 × 10^4 ^(HL-60) or 1 × 10^5 ^(MOLT-4) per well in 96-well cell culture plates and incubated with respective drug solutions of different concentrations for 96 hr and processed. All vehicle controls contained same concentration of DMSO. The plate was read in a microplate reader at 540 nm. Curvefit software was used to calculate the IC_50 _values. IC_50 _value < 10 μM is considered as active as per National Cancer Institute (NCI), USA, protocol.

**Table 1:** In vitro screening in human tumor cell lines

	IC_50 _value (μM)*		
	
Compound	Lymphoma	Leukemia	
		
	U-937	HL-60	MOLT-4
**1a**	25.3	15.7	-
**1b**	37.5	19.3	-
**1c**	28.4	32.5	-
**1d**	1.4	0.7	4.2
**1e**	24.6	26.9	-
**1f**	32.7	17.6	-
**1g**	29.2	57.6	-
**1 h**	36.9	26.0	-
**1i**	1.0	0.8	6.0
**1j**	18.6	39.9	-
Doxorubicin	-	-	11.0
5-FU	4.7	266	-
Cis-Platin	3.2	7.0	-
BCNU	12.3	30.5	-
Hydroxyurea	115	204	-

Cytotoxicities of test compounds **1d **and **1i **were further evaluated against 11 other human tumor cell lines by SRB assay method [10] as stated in Table 2. Growth inhibition value 50% or more at 1 × 10^-5^M is considered as active. Established anticancer drugs such as doxorubicin, 5-FU, cis-platin, BCNU, hydroxyurea, paclitaxel and mitomycin C were used in parallel for comparison as indicated in the respective Table 1 and 2.

**Table 2:** In vitro screening in human tumor cell lines

Compound	Conc. (M)	Breast	Neuroblastoma		Liver	Colon				Lung	Prostate	
							
		MCF-7	IMR-32	SK-N-SH	Hep-2	502713	Colo-205	HCT-15	SW-620	A549	DU-145	PC-3
Growth inhibition (%)*												
**1d**	1 × 10^-6^	0	32	0	-	-	8	4	-	0	-	-
	1 × 10^-5^	23	69	41	-	-	64	29	-	23	-	-
**1i**	1 × 10^-6^	15	71	45	5	43	26	12	34	-	34	0
	1 × 10^-5^	24	39	89	26	84	23	24	56	-	53	51
5-FU	1 × 10^-5^	30	-	66	-	45	-	-	26	-	-	-
Paclitaxel	1 × 10^-6^	-	72	-	-	-	-	76	62	62	-	-
Mitomycin C	1 × 10^-6^	-	-	50	-	-	43	-	71	-	58	46
	1 × 10^-5^	60	-	85	49	-	-	70	-	-	-	-
Doxorubicin	1 × 10^-6^	37	-	51	64	-	-	-	-	-	-	-
	1 × 10^-5^	-	-	-	-	-	-	47	-	-	-	70

### Effect on PBMC

PBMC was isolated from heparinized venous blood obtained from healthy human volunteer by Ficoll-Paque (Histopaque 1077, Sigma-Aldrich Corporation, St. Louis, MO, USA.) density gradient centrifugation as per standard procedure [11]. PBMC (1 × 10^5 ^cells/well) were cultured in complete RPMI-1640 media as usual and incubated with compounds **1d **and **1i **for 48 hr followed by MTT assay. IC_50 _values were calculated using Curvefit software.

### Analysis of cell cycle

**Figure 2:**
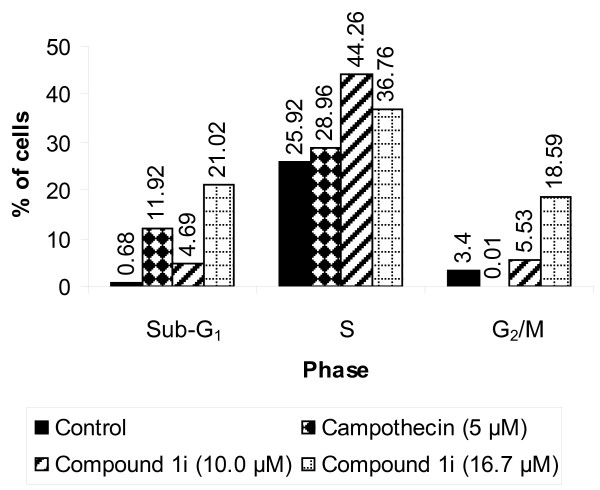
**Flow cytometric assessment of cell cycle of MOLT-4 cells (1 × 10^6^/ml) treated in vitro with compound 1i (10.0 and 16.7 μM) for 24 h or camptothecin (5.0 μM) for 3 hr as reference**. Treatment with compound **1i **resulted in marked rise in sub-G_1_, S and G_2_/M fractions suggesting apoptosis and mitotic delay, respectively.

The effect of compound **1i **on different phases of cell cycle of MOLT-4 was explored by flow cytometry [12]. In brief, 1 × 10^6 ^MOLT-4 cells were incubated with compound **1i **(10.0 and 16.7 μM) for 24 hr and camptothecin (5 μM) for 3 hr. The cells were next washed twice with ice-cold phosphate buffered saline (PBS), harvested, fixed with ice-cold PBS in 70% ethanol, and stored at -20°C for 30 min. After fixation, the cells were incubated with RNase A (Sigma-Aldrich Corporation, St. Louis, MO, USA, 0.1 mg/ml) at 37°C for 30 min, stained with propidium iodide (Sigma-Aldrich Corporation, St. Louis, MO, USA, 50 μg/ml) for 30 min on ice in dark and analyzed for DNA content using BD-LSR Flow cytometer (Becton Dickinson, USA). Data were collected in list mode on 10,000 events and analyzed using Mod Fit 2.0 software (Figure 2).

### Assessment of apoptosis

**Figure 3:**
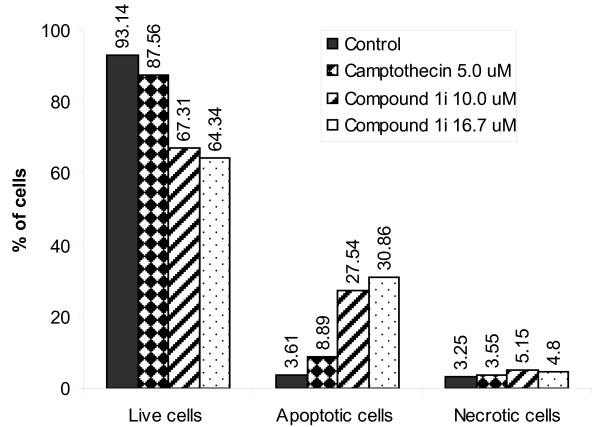
**Induction of apoptosis by compound 1i (10.0 and 16.7 μM) and camptothecin (5.0 μM) in MOLT-4 cells (1 × 10^6^/well)**. Live, apoptotic and necrotic cells were analyzed by flow cytometry after staining with annexin V-FITC and propidium iodide.

**Figure 4:**
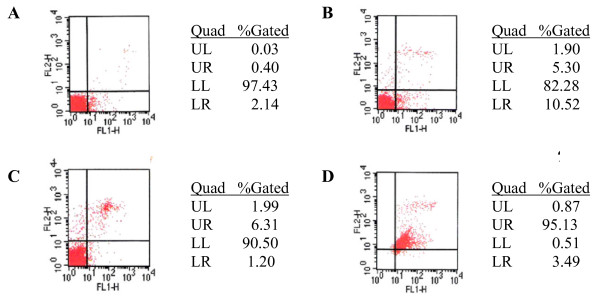
**Analysis of apoptosis induced by compounds in HL-60 cells (5 × 10^5^/well) by flow cytometry using annexin V-FITC and PI**. Quadrant analysis of fluorescence intensity of gated cells in FL-1 (annexin V-FITC) and FL-2 (PI) channels was from 10,000 events. A: Stained control; B: Camptothecin (10 μM); C: Cisplatin: (10 μM); D: **1i **(10 μM).

Annexin V-FITC/PI double staining method was followed [13] for the assay in MOLT-4 cells (1 × 10^6^/well, 6-well plate) after incubation of the cells with 10.0 and 16.7 μM of compound **1i **and 5 μM of camptothecin for 6 hr at 37°C (Figure 3). Similar assay was conducted in HL-60 by using another apoptosis detection kit (BD Biosciences Pharmingen, San Diego, USA). For this, HL-60 cells (5 × 10^5^/well) were treated for 24 hr with compounds **1i**, camptothecin and cis-platin (10 μM concentration each). Cells were processed and stained with Annexin V-FITC/PI according to the manufacturer's instructions and analyzed on a FACScan flow cytometer (Becton Dickinson, USA) using Cell Quest software at two wavelengths 515 and 639 nm. Vehicle (DMSO) treated unstained and stained [annexin V-FITC/PI] cells were used as controls (Figure 4).

### Measurement of caspase-3/6 activities

**Figure 5:**
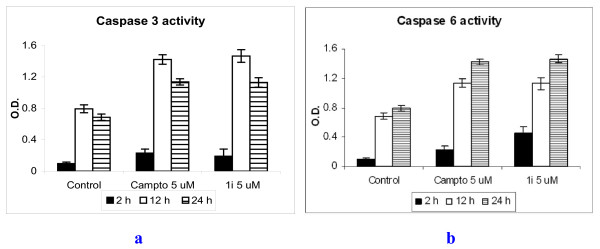
**a Caspase 3 and b caspase 6 activities in MOLT-4 cells (2 × 10^6^/ml) treated with 5.0 μM compound 1i and 5.0 μM of camptothecin (reference) for 2-24 h in vitro**.

The activities of caspase-3 and caspase-6 in MOLT-4 cells (2 × 10^6^/ml) following incubation with compound **1i **(3.3 - 16.7 μM) and camptothecin (5 μM) for variable periods were measured by using respective colorimetric assay kit (R&D Systems, USA). Blank cell lysate control was also included. Enzyme-catalyzed release of pNA was monitored using a microplate reader at 405 nm (Figure 5A and 5B).

### Cell morphological and ultra structural assessment

**Figure 6:**
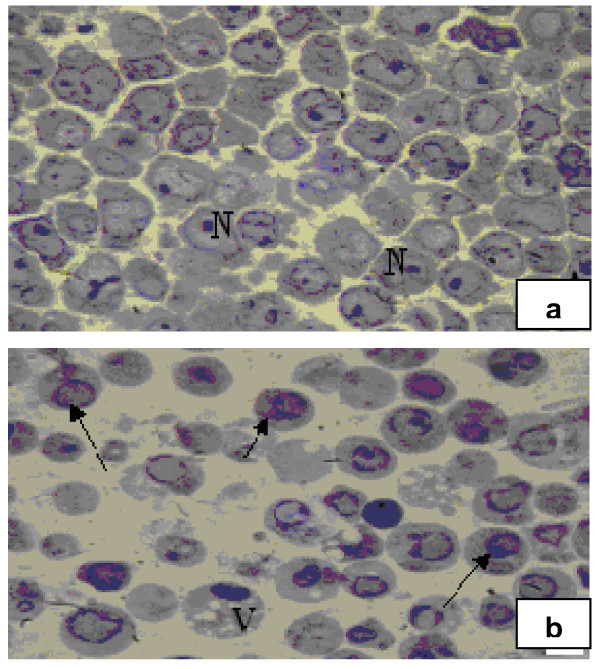
**Photomicrographs of a control and b compound 1i treated MOLT-4 cells exposed to 10 μM of compound for 36 h in vitro**. Compared with control cells with large nuclei (N) and prominent nucleoli, treated cells displayed marginalized chromatin material (arrow) and cytoplasmic vaculation (V), the hallmark of apoptosis. (Mag. 1000×).

**Figure 7:**
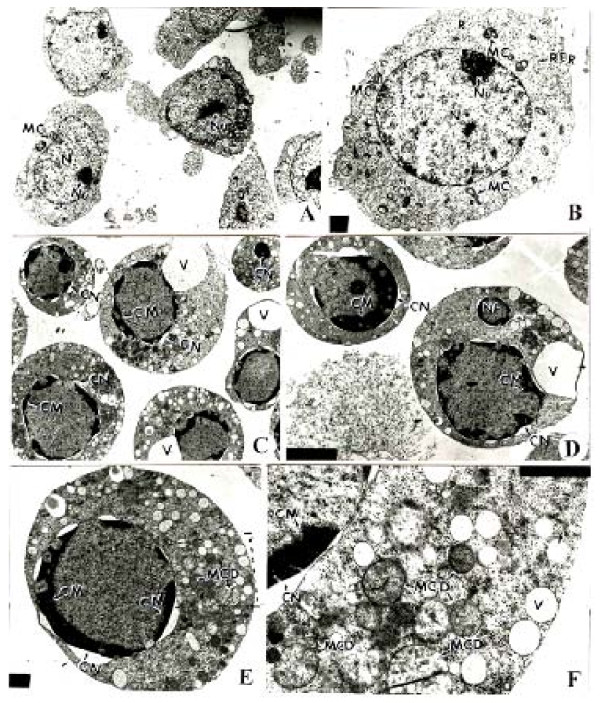
**TEM of control and compound 1i treated (10 μM for 36 h) MOLT-4 cells showing internal ultra structure**. The control cells show nucleus (N) with finely dispersed chromatin material and a nucleolus (Nu). The mitochondria with cristae (MC) and ribosomes (R) are seen (Figure A-B). The treatment causes chromatin marginalization (CM), condensation of the nucleus (CN), and vacuolization (V) in the cytoplasm (Figure C-F).

MOLT-4 cells were incubated with compound **1i **(10 μM) in DMSO for different time periods. Control cells received DMSO only (< 0.5%). Treated and control cells were washed in PBS, centrifuged at 1500 rpm for 10 min. Pellets were divided into 1 mm^3 ^pieces and fixed immediately in 2.5% glutaraldehyde in 0.1 M phosphate buffer (pH 7.2) for 2 hr at 4°C, post-fixed with 1% OsO_4 _in the same buffer for 2 hr, dehydrated with acetone, cleared in propylene oxide and embedded in Epon-812 [14]. Semithin (1 μm) sections were cut, stained with toluidine blue and morphology of treated cells was observed [14] at different times under light microscope [Olympus, Japan]. Photomicrographs were taken with Olympus Digital Camera (C4000) (Figure 6). Ultrathin sections of silver color (60-90 nm) were cut on a LKB ultramicrotome IV, mounted on copper grids and stained with uranyl acetate and lead citrate. The sections were viewed and photographed in a JEOL-100CXII electron microscope at 60 kV (Figure 7).

**Figure 8:**
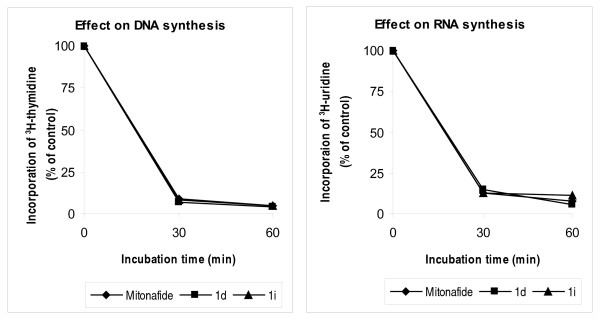
**Effects of compound 1d, 1i and Mitonafide at 8 μM concentration each on the synthesis of DNA and RNA in S-180 tumor cells**. Results are expressed as percentage of ^3^H-thymidine and ^3^H-uridine incorporation in untreated control cells.

### ^3^H-Thymidine and ^3^H-Uridine incorporation in S-180 cells in vitro

S-180 tumor cells maintained in vivo in Swiss albino mice were used for incorporation of ^3^H-thymidine and ^3^H-uridine (specific activity 1.0 mCi/ml each, obtained from Board of Radiation and Isotope Technology, Mumbai, India) following treatment with 8 μM concentration of compounds **1d **and **1i **as described earlier [15]. Mitonafide at the same concentration was used for comparison.

#### Abbreviations used

MTT: [3-(4,5-dimethylthiazol-2-yl)-2,5-diphenyl-2H-tetrazolium bromide]; SRB: sulphorhodamine B; DMSO: dimethylsulfoxide; S-180: Sarcoma-180; PBMC: peripheral blood mononuclear cells; IC_50_: 50% inhibitory concentration; 5-FU: 5-Fluorouracil; BCNU: bis(2-chlorothyl)nitrosourea.

### Statistical analysis

Values were recorded as the mean ± S.E.M. (standard error mean) of three experiments. Experimental results were analyzed by Student's t-test. P < 0.05 was considered as the level of significance for values obtained for treated groups compared with control group.

## Results

### Cytotoxicity screening

In vitro screening of compounds **1a-j **against U-937 and HL-60 revealed that compounds **1a-c**, **1e-1h **and **1j **did not show appreciable activity as their IC_50 _values were above 10 μM. Compounds **1d **and **1i **having IC_50 _values in the range of 0.7 and 6.0 μM in U-937, HL-60 and MOLT-4 were found to be cytotoxic (Table 1). The IC_50 _values of compounds **1d **and **1i **were much less than that of doxorubicin, 5-FU, cis-platin, BCNU and hydroxyurea used as standards (Table 1) suggesting greater antitumor properties in compounds **1d **and **1i**. In view of this, compounds **1d **and **1i **were selected for further screening in a battery of human tumor cell lines. The results summarized in Table 2 revealed that compound **1d **has elicited significant growth inhibition in two (IMR-32 and COLO-205) out of six cell lines used while compound **1i **elicited significant growth inhibition in five (SK-N-SH; 502713, SW-620, DU-145 and PC-3) out of ten cell lines tested. It appears that compound **1i **is the most active member.

### In vitro toxicity screening in PBMC

Compounds **1d **and **1i **showed high IC_50 _values of 698 and 273 μM respectively against human PBMC in vitro suggesting that these compounds were devoid of significant cytotoxicity against normal cells.

### Effect on cell cycle

MOLT-4 cells exposed to 10.0 and 16.7 μM of compound **1i **for 24 hr exhibited increase in sub-G_1 _fraction which may comprise of both apoptotic cells and cell debris implying up-regulation of cell death machinery. The effect was much more for the higher concentration of the compound. For instance, the sub-G_1 _fractions of control and camptothecin-treated cells were 0.68% and 11.92% respectively whereas the same were 4.69% and 21.02% for compound **1i **at the low and high concentrations (Figure 2). This might indicate a dose dependant increase in apoptosis of MOLT-4 cells inflicted by compound **1i**. The cell cycle analysis also showed accumulation of treated cells in S and G_2_/M phases. Increase in S phase fraction could be due to stimulation of DNA synthesis or delay in movement of cells from S to G_2_/M phase. Concomitant rise in G_2_/M fraction indicates delay in exit of daughter cells from the mitotic cycle. Therefore the findings suggest delayed turnover of cells leading to reduction of tumor cell number.

### Analysis of apoptosis in MOLT-4 and HL-60 cells by Annexin V-FITC/PI double staining method

MOLT-4 and HL-60 control and treated cells were stained with annexin V-FITC/PI and gated into LR (Lower Right) and UR (Upper Right) quadrants. Cells in LR and UR were considered as early apoptotic (annexin^+^/PI^-^) and late apoptotic (annexin^+^/PI^+^) respectively. Extent of apoptosis was expressed as the sum total of the percentages in LR and UR quadrants. Cells in LL (Lower Left) and UL (Upper Left) quadrants were considered live and necrotic respectively. Apoptosis induced by compound **1i **was compared with that of camptothecin (Figure 3) and camptothecin and cis-platin used as standards (Figure 4). Apoptosis recorded in untreated control MOLT-4 and HL-60 cells were 3.61% and 2.54% respectively.

In MOLT-4, total apoptosis exhibited by camptothecin at 5 mM concentration was 8.89%. In contrast compound **1i **at 10.0 and 16.7 mM concentrations was effective in inducing 27.54% and 30.86% apoptosis respectively. The necrotic cell populations for compound **1i **at these doses were 5.15% and 4.80% respectively (Figure 3).

In HL-60, compound **1i **induced 98.62% apoptosis at a dose of 10 μM (LR 3.49%, UR 95.13%). This is in contrast to 15.82% and 7.51% apoptosis respectively induced by camptothecin and cisplatin at the same dose. Thus compound **1i **was more effective than standards in inducing apoptosis in HL-60 (Figure 4).

### Activation of caspases

Treatment of MOLT-4 cells with compound **1i **was associated with marked increase in caspase-3 as well as caspase-6 activities that confirm the apoptotic mode of cell death. Up-regulation of caspase-3 by compound **1i **was maximum at 5.0 μM concentration at 12 hr post-treatment (Figure 5a) while caspase-6 activity was highest also at 5.0 μM concentration at 24 hr post-treatment (Figure 5b). Similar activations were produced by camptothecin at 5.0 μM concentration (Figure 5a-b).

### Cell morphological and ultra structural assessment

The morphology of MOLT-4 cells treated with compound **1i **at 5 and 10 μM was monitored by light microscopy at different time points. The number of apoptotic cells increased with higher concentration of the compound and longer incubation period. Figure 6b represents the characteristic morphology of apoptotic cells following 36 hr of incubation at 10 μM concentration. Marginalization of chromatin material accompanied by cell shrinkage, nuclear condensation/fragmentation and formation of cytoplasmic vacuoles, considered as hallmark of apoptosis, were clearly visible. Control cells showed large sized nuclei having nucleoli (Figure 6a).

In transmission electron microscopy, MOLT-4 control cells (Figure 7a-b) exhibited a high nucleocytoplasmic ratio and the nucleus had a finely dispersed chromatin with nuclear pores. The nucleoli were clearly visible in most of the cells. The mitochondria with cristae (MC) in various size and shape (oval and elongated), rough endoplasmic reticulum and ribosomes were seen. MOLT-4 cells treated with 10 μM of compound **1i **for 36 h revealed damaged mitochondrial cristae and highly reduced rough endoplasmic reticulum suggesting apoptosis (Figure 7c-f). No inflammatory changes in nuclei and cytoplasm coupled with absence of breakage in plasma membrane ruled out the possibility of necrotic events. Vacuolization was also seen in treated cells. Literature survey also revealed similar observations [16,17].

### Inhibition of DNA/RNA synthesis in S-180 tumor cells in vitro

Since compound **1d **and **1i **have structural similarity with mitonafide, studies were conducted to ascertain whether drug-induced tumor growth inhibition was also due to the inhibitory effect of these compounds on nucleic acid synthesis. Accordingly ^3^H-thymidine and ^3^H-uridine incorporation by S-180 cells collected from untreated tumor bearing mice was measured after treating the tumor cells in vitro. The untreated S-180 cells demonstrated an almost linear pattern of ^3^H-thymidine and ^3^H-uridine incorporation over a period of 60 min. Exposure of tumor cells to test compounds at the concentration of 8 μM resulted in gradual and marked inhibition of ^3^H-thymidine and ^3^H-uridine incorporation comparable to that of mitonafide at the same concentration (8 μM). After 1 hr of incubation with compound **1d **and **1i **^3^H-thymidine incorporation was declined by 96% and 95% respectively against 95% reduction by mitonafide exposure. Thus the compounds showed remarkable inhibitory effect on DNA synthesis. Inhibition of RNA synthesis, in contrast was less spectacular as inhibition of ^3^H-uridine was 92%, 94% and 89% for mitonafide, compound **1d **and **1i **respectively (Figure 8).

## Discussion

The nature and position of a substituent in a molecule are known to play important roles in deciding its antitumor property. The present study has shown that out of the five different substituents (R = H, 6-Br, 6-Cl, 6-NO_2_, 5-NO_2_) present in the aromatic ring portion of substituted N-(hydroxyalkyl)naphthalimide moiety, the 6-NO_2 _substituent is crucial in exercising the antitumor activity. This is in agreement with our earlier finding in other (chloroalkyl) naphthalimide compounds wherein we found 6-nitro-2-(3-chloropropyl) naphthalimide as the most active antitumor agent in that series [7].

Compound **1i **that showed most pronounced antitumor activity interfered with S and G_2_/M phases of cell cycle of MOLT-4 cells. As a preparatory step towards cell division, a cell duplicates its DNA in S phase of cell cycle. Thus, interference of S phase by compound **1i **as observed in flow cytometric measurements, suggests that it affects DNA duplication process of tumor cell before mitosis. This possibility was confirmed in S-180 cells in which compound **1i **inhibited ^3^H-thymidine incorporation into DNA, implying suppression of DNA synthesis. Moreover, it inhibited ^3^H-uridine uptake, indicating concomitant inhibition of RNA synthesis. Taken together, the results suggest that inhibition of DNA and RNA might have played a role in mediating the antitumor effect of compound **1i**.

Delay in exit from G_2_/M, the final phase of cell cycle, was another flow cytometric observation in compound **1i **treated MOLT-4 cells. A situation like this develops when there is defect in DNA damage repair, spindle attachment with centromeres and polymerization of spindle microtubules [18]. In view of these reports, it appears that the compound has adverse effect on the mitotic apparatus causing up-regulation of the spindle checkpoint control leading to delayed mitotic exit of daughter cells. It is known that vinca alkaloids [19] and paclitaxel [20] mediate their antitumor effects by interfering with spindle microtubules. Compound **1i **may act in a similar fashion like them.

Induction of apoptosis or programmed cell death is a common mechanistic pathway of several antitumor agents [21]. Compound **1i **has exerted its antitumor action by this pathway as well. This is evident from sharp rise in sub-G_1 _fraction, light and electron microscopic studies showing morphological imprints of apoptosis and marked increase in caspase 3 and 6 in treated cells. Apoptosis is controlled by a diverse range of cell signals which may originate intracellularly via the mitochondria or extracellularly via death receptors on cell membranes. These two pathways of signals converge and form a common irreversible execution phase mediated by caspase 3 and 6. Whether the pro-apoptotic signal elicited by compound **1i **followed the intrinsic (mitochondrial) or extrinsic (death receptor) pathway is not clearly understood. However, extensive damage of mitochondrial cristae in treated cells, as observed in ultrastructural study, favours mitochondrial pathway. Like the present finding, induction of apoptosis by many naphthalimides including amonafide and amonafide analogs has been reported [22,23].

In essence, the present study demonstrated significant antitumor activity by compound **1i **against murine S-180 tumor cells and a panel of human tumor cell lines in vitro and the effect was mediated by inhibition of cell proliferation and up-regulation of programmed cell death. Since the compound did not elicit any cytotoxicity against normal human PBMC, it holds promise for further development as a potential antitumor agent.

## Competing interests

The authors declare that they have no competing interests.

## Authors' contributions

US and AKS designed and co-coordinated the study at the respective Institutes [CNCI & IIIM]. AM and SD prepared the compounds & have carried out various biological experiments. MS carried out in vitro cytotoxicity screening in human tumor cell lines. DMM participated in the design of the study and performed cell cycle analysis. PRS performed cell morphological and ultra structural assessment. SKS carried out assessment of apoptosis and measurement of caspase-3/6 activities. AKS has helped to draft the manuscript. US has analyzed the data and prepared the manuscript. All authors have read and approved the final manuscript.
